# A convolutional neural network segments yeast microscopy images with high accuracy

**DOI:** 10.1038/s41467-020-19557-4

**Published:** 2020-11-12

**Authors:** Nicola Dietler, Matthias Minder, Vojislav Gligorovski, Augoustina Maria Economou, Denis Alain Henri Lucien Joly, Ahmad Sadeghi, Chun Hei Michael Chan, Mateusz Koziński, Martin Weigert, Anne-Florence Bitbol, Sahand Jamal Rahi

**Affiliations:** 1 Laboratory of the Physics of Biological Systems, Institute of Physics, École polytechnique fédérale de Lausanne (EPFL), Lausanne, Switzerland; 2 Institute of Bioengineering, School of Life Sciences, École polytechnique fédérale de Lausanne (EPFL), Lausanne, Switzerland; 3 Computer Vision Laboratory, École polytechnique fédérale de Lausanne (EPFL), Lausanne, Switzerland

**Keywords:** Bioinformatics, Software, Image processing

## Abstract

The identification of cell borders (‘segmentation’) in microscopy images constitutes a bottleneck for large-scale experiments. For the model organism *Saccharomyces cerevisiae*, current segmentation methods face challenges when cells bud, crowd, or exhibit irregular features. We present a convolutional neural network (CNN) named YeaZ, the underlying training set of high-quality segmented yeast images (>10 000 cells) including mutants, stressed cells, and time courses, as well as a graphical user interface and a web application (www.quantsysbio.com/data-and-software) to efficiently employ, test, and expand the system. A key feature is a cell-cell boundary test which avoids the need for fluorescent markers. Our CNN is highly accurate, including for buds, and outperforms existing methods on benchmark images, indicating it transfers well to other conditions. To demonstrate how efficient large-scale image processing uncovers new biology, we analyze the geometries of ≈2200 wild-type and cyclin mutant cells and find that morphogenesis control occurs unexpectedly early and gradually.

## Abstract (web summary)

Current cell segmentation methods for *Saccharomyces cerevisiae* face challenges under a variety of standard experimental and imaging conditions. Here the authors develop a convolutional neural network for accurate, label-free cell segmentation.

## Introduction

**Fig. 1:**
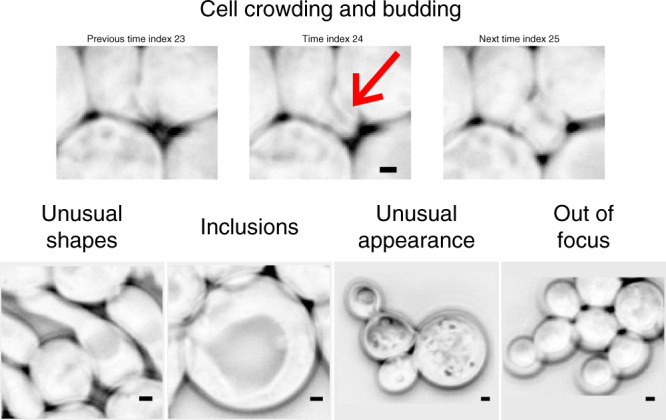
Challenging cases for the segmentation of yeast images. The red arrow points to a difficult-to-see new bud that appears in a timelapse movie. Scale bar: 1 *μ*m. Phase contrast images inverted for better visualization.

Budding yeast is an important model organisms in genetics, molecular biology, systems biology, and synthetic biology. Almost all current segmentation methods for yeast images^1–9^ rely on classical image processing techniques^10^ such as thresholding, edge detection, contour fitting, and watershed. However, for many experiments, the segmentations produced by these tools require frequent user interventions. Common challenges for yeast image segmentation include cell crowding, irregular shapes, transparent inclusions (e.g., vacuoles), unusual visible features, budding events, and imperfect focus during imaging (Fig. 1).

CNNs have established themselves in recent years as efficient and powerful computational models for segmentation tasks^11^. CNNs replace sophisticated classical image processing algorithms with neural-network based models which are trained on a sufficiently large and diverse set of examples^12^. A key advantage of CNNs over non-learning based approaches is that in order to improve the predictions for new cases or conditions, fundamentally new ideas are not needed. In principle, new cells or conditions that the system performs poorly on only need to be included in sufficient numbers in the training set. We demonstrate this advantage with clb1-6Δ mutants that create filamentous buds.

Despite the importance of *S. cerevisiae* as a model organism, to the best of our knowledge, gold-standard image and segmentation data sets for yeast or CNNs trained on such data sets do not exist. Training data in the form of manual annotations of cell masks is expensive and labor-intensive to generate, especially if it needs to include mutants, which are important for many laboratories. To segment accurately, human annotators need experience with yeast cell images. Furthermore, it is not widely known which of the many available artificial neural network architectures is suited best, what the disadvantages of each are, and how they can be mitigated.

Previous work demonstrated that a CNN can segment yeast images better than competing methods under very low light conditions^13^. However, the training set was focused on the specific challenge of very low light levels. YeastSpotter, a CNN for yeast image segmentation based on the Mask-RCNN architecture, was not trained on yeast images but mostly on human cell nuclei^14^. Thus, it is not surprising that many images of yeast cells cause it to make mistakes (see “Comparison to other methods and benchmarking”). The bright-field images of diploid yeast cells published by Zhang et al.^15^ contain in-focus and substantially out-of-focus cells in the same field of view, with only the in-focus cells segmented; it is unclear how well a neural network trained on this data set could detect out-of-focus buds or segment images that are slightly out of focus as in Fig. 1. The web resource YIT^16^ contains high-quality bright-field and phase contrast images of wild-type yeast cells but only the cell centers are annotated, not the borders.

Beyond yeast, the approach of DeepCell^17^, which was applied to bacterial and mammalian cells and which inspired ours, has the drawback of requiring an additional fluorescent channel for segmentation, which we seek to avoid. Experiments may need all available fluorescent channels for measurements or may involve optogenetic constructs.

Here, we present a large, diverse data set for yeast segmentation and an easy-to-train CNN, which we call YeaZ (pronounced: y-easy). A Python-based graphical user interface (GUI) can be used to apply the CNN to images in a user-friendly manner, to visualize the images and the segmentation masks, to apply the bipartite matching algorithm for tracking^8^, and to correct potential mistakes. In order to avoid the need for fluorescent nuclei marking the cell interiors as in the DeepCell method^17^, we seed cells based on peaks of the distance transform and perform a “cell-cell boundary test” to remove erroneous borders^18–21^. Using the YeaZ CNN to measure the cell geometry of hundreds of wild-type and cyclin mutant cells, we find differences in elongation which indicate that the mitotic cyclin *CLB2* controls cell morphology unexpectedly early and gradually. To assess the suitability of the YeaZ CNN without installing any software, images can be submitted to a website for segmentation, accessible under http://www.quantsysbio.com/data-and-software. Users are invited to submit challenging images for inclusion in the training set, which thus will expand with time and improve the CNN.

## Results

### Data set

**Fig. 2:**
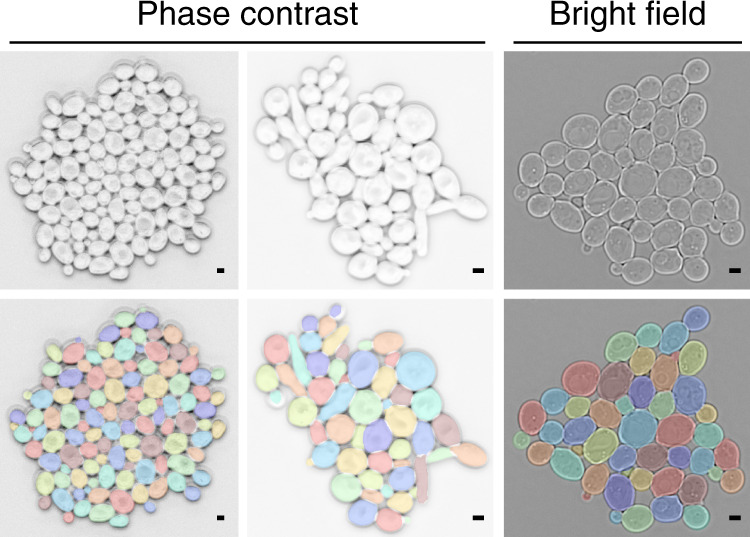
Overview of the YeaZ training data set. Shown are examples of raw images acquired with phase contrast or bright-field microscopy (upper row) and corresponding manual annotations (lower row). Phase contrast image inverted for better visualization. Scale bar: 2 *μ*m.

We segmented >8500 budding yeast cells of strain background W303, recorded by phase contrast microscopy, semi-manually using a custom image processing pipeline (Fig. 2, Supplementary Table 1). In total, this resulted in 384 images (saved in multi-layer tif files) and corresponding manual annotation masks, which were checked by 1–2 other people. The set includes normally growing, pre-Start (clnΔ) arrested, filamentous G1/S (clb1-6Δ) arrested, metaphase (cdc20Δ) arrested, and DNA damaged cells, some of which are shown in Fig. 1. Cells were often in large colonies, in which even by eye, cell borders can be difficult to ascertain. Older and bigger cells contained large, transparent inclusions, likely vacuoles, which many classical image segmentation techniques fail to ignore because their edges look like cell borders. Potentially sick cells with strange visible features were included (Fig. 1). Cell sizes varied widely from about 0.4 to 80 *μ*m^2^ (mean wild-type size ≈ 16 *μ*m^2^). We annotated barely visible buds. Some images were sufficiently out of focus for cells to develop a second light ring around them, which makes identification of the cell edge difficult for many methods (Fig. 1).

Using the following trick, we segmented another >1700 cells recorded by bright-field microscopy (Fig. 2, Supplementary Table 2): We took images of the same scene of wild-type cycling cells by bright-field and by phase contrast microscopy in rapid succession. Then, we used the YeaZ CNN to segment the phase contrast images efficiently and transferred the segmentation masks to the bright-field images. However, for the rest of our work, we did not use the bright-field segmentations but are making the data available to the community.

Data augmentation artificially increased the size of the training set even further by rotating, flipping, shearing, enlarging, or shrinking, as well as dimming or brightening the images for training the CNN (see “Methods”).

### Convolutional neural network (CNN)

We evaluated three well-known convolutional neural network architectures: U-Net^22^, Mask-RCNN^11^, and Stardist^23^. We chose U-Net and trained it to distinguish pixels belonging to cell bodies (mapped to 1) from background or cell–cell border pixels (mapped to 0). We did not further distinguish between background and cell-cell border pixels^17^ because the two were often difficult to discriminate unambiguously during the annotation of the training set and because even without this differentiation the resulting CNN was highly accurate. We decided against Mask-RCNN because of artefactual cut-offs of the identified cell regions, presumably due to the method’s rectangular bounding boxes. Stardist alleviates this problem by approximating cell borders with star-convex polygons. Although such a representation is likely appropriate for the typical round shapes of wild-type cells, it is ill-suited for elongated and filamentous cell shapes such as of clb1-6Δ cells.

### Segmentation steps

**Fig. 3:**
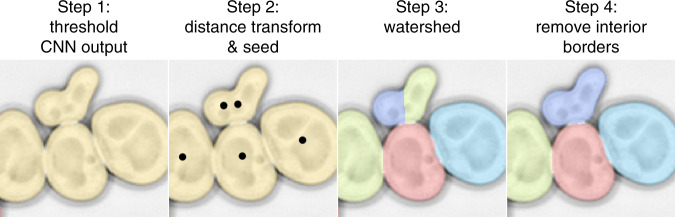
Steps to segmentation: (1) threshold the CNN output, (2) find the peaks of the distance transform (=seeds), (3) watershed, (4) remove erroneous interior borders using a cell–cell boundary test. Phase contrast image inverted for better visualization. Image from ref. ^16^, scale bar unknown.

**Fig. 4:**
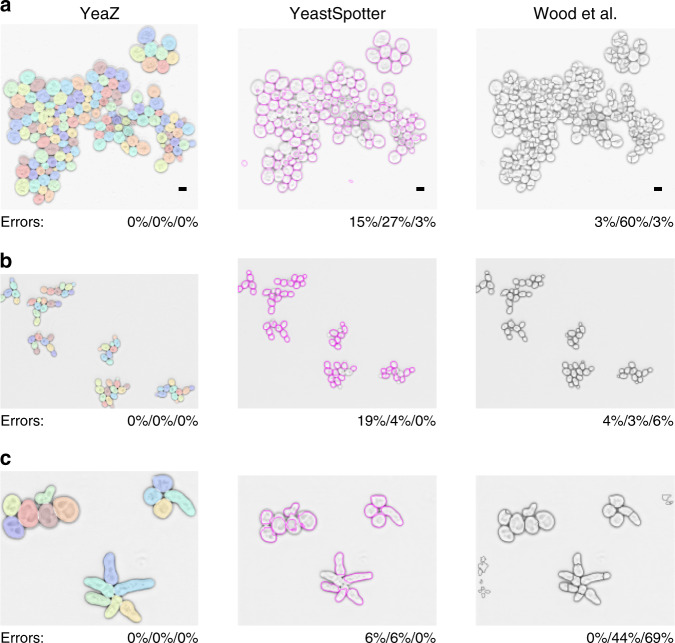
Comparison of YeaZ with YeastSpotter and Wood et al.^9^. **a** Image recorded by us but not included in the training set. **b** Image from ref. ^16^ showing cycling cells. **c** Image from ref. ^16^ showing pheromone-arrested cells. The error values represent the fraction of missed cells/of bad contours/and of spurious cells. Phase contrast images are inverted for better visualization. Scale bar in row **a**: 5 *μ*m. Images in rows (**b**, **c**) from ref. ^16^, scale bar unknown.

The CNN assigns to each pixel a score from 0 (border- or background-like) to 1 (cell-like). These continuous scores are turned into a segmented image by the following steps (Fig. 3):

1. Initial cell-versus-non-cell classification: A threshold of 0.5, arbitrary but intuitive, is used to distinguish putative cell pixels from the rest. This step already identified most cell bodies as distinct from one another in our images. However, some cells were connected by bridging putative cell pixels, which is why the following steps were needed.
2. Find a point inside each cell: For each putative cell pixel, the distance transform (the shortest Euclidean distance to a border/background pixel) is computed. Pixels at which the distance transform has a maximum within a radius of 5 pixels (≈0.5 *μ*m) identify putative interior points of cells. This step successfully identified one or more points in each cell in our images. (To detect very small buds, we lowered this threshold, see “Comparison to other methods and benchmarking”.)
3. Assign a putative cell to each interior point: each peak of the distance transform is used as a seed for the watershed method, which assigns regions of pixels to each peak. These regions are the putative cells.
4. Remove erroneous cell boundaries: since the distance transform may yield more than one point inside each cell, e.g., for a dumb-bell shaped cell, a real cell may be erroneously subdivided into multiple regions by the watershed procedure. This is a well-recognized problem in image segmentation^12,18–21^ and could be circumvented, for example, by a fluorescent nuclear marker specifying a unique interior point. To avoid the requirement for an additional channel, we devised the following cell-cell boundary test: For all pairs of putative cells, we evaluate whether the pixels on the boundary are too cell-like; if the average CNN score for the top 3/4 of boundary pixels (bottom 1/4 is ignored because an erroneous boundaries will touch real boundaries at their two ends) is above 0.99, i.e., very cell-like, this boundary is likely erroneously subdividing a real cell. In that case, the two regions separated by the erroneous boundary are merged. This strategy fixed all cases of split cells that we encountered, which, for example, occurred for 10% of cells in Fig. 4 (top). We did not observe that any cells were joined erroneously.

We introduced a small number of parameters in the above steps without fine-tuning because the results did not require it (0.5 CNN-score threshold, five pixel distance-transform threshold, 0.99 average CNN-score threshold on 3/4 of boundary pixels), as demonstrated in “Comparison to other methods and benchmarking”.

### Tracking

The tracking algorithm is similar to the one in CellStar^8^. Cells are matched between two consecutive frames. For each time point *t* and each cell *i*, the center of mass and the area are calculated (*x*_*i*_(*t*), *y*_*i*_(*t*), *A*_*i*_(*t*)). The mean over all cells at time *t* is subtracted and the resulting triplets are rescaled to normalize the variances to (3, 3, 1). (We observed that weighting in favor of the position makes the algorithm work better.) The actual tracking step is performed by bipartite graph matching, finding pairings that minimize the summed Euclidean distances between the normalized triplets. This can be done efficiently by the Hungarian algorithm^24,25^.

We assessed the quality of this tracking method with a 75-frame timelapse recording from our training set that starts with 11 cells and ends with 49 cells. Of the 1903 frame-to-frame correspondences that had to be found, four were erroneous. All of these mistakes occurred between two time points when two cells floated away and two new buds appeared (Supplementary Fig. 1). Thus, the tracking method appears to be highly reliable but we did not evaluate it further since tracking is not the focus of this work.

### Comparison to other methods and benchmarking

The convolutional neural network approach has at least two inherent advantages over non-machine learning approaches: While diverse and potentially difficult to analyze, budding yeast cells may only have a range of shapes and visible features. Our large, diverse data set is well suited to cover this range and enables the neural network to interpolate between shapes it has already been trained with in order to segment new images. Furthermore, should a particular condition or cell type not yield satisfactory segmentation results, the addition of a number of new examples in principle suffices to expand the capabilities of the neural network, as we demonstrate for clb1-6Δ cells.

Ideally, to compare YeaZ to other methods, we would use a gold-standard segmentation benchmark. However, we could not find such a data set, which is in part why we believe our data set of segmented images will be useful to the community. Instead, we proceed as in a comprehensive comparison of segmentation methods performed previously^16^. We begin by focusing on three images: one image of moderate complexity from our timelapse recordings that was not included in the training set (a) and two images of budding yeast cells included in the prior comparison^16^ containing cycling (b) or pheromone-arrested (c) wild-type cells, respectively. Images (b) and (c) represent the last time points in two timelapse recordings (data sets 9 and 10 in ref. ^16^) and thus are the most complex images of the series, containing the largest number of cells. Together, the three images cover three important situations: a crowded scene (a), a relatively sparse scene (b), and new shapes (c) not included in our training set.

We chose for our comparison the newest segmentation method we found published, by Wood et al.^9^, and the only other available neural network for yeast segmentation YeastSpotter^14^, which was, however, not trained on yeast cell images. Wood et al.’s method has at least 16 parameters; we varied min_cell_size, max_cell_size, min_colony, clean_BW, and size_strel_bg_2 away from the default values to improve the results. Since the method by Wood et al.^9^ compares favorably with other published methods^16^ and given the stark differences in the segmentation qualities we observed, we confined ourselves to these two comparisons.

The results of the YeaZ segmentation are perfectly accurate for all three images (Fig. 4). No tweaking of our parameters was necessary except to adjust roughly the pixel equivalent of the 0.5 *μ*m threshold for the distance transform to the larger pixel sizes in (b) and (c). Close inspection of the results revealed no missed cells, no missed buds, no errors in the boundary assignments, and no false cells. Given the many differences between our strains and conditions and those of the images from ref. ^16^, this exemplifies the transferability of the YeaZ CNN.

In order to compare the results in a way that is useful for the typical user, we scored the output of the other methods by counting the number of cells that were missed by the segmentation, that were segmented clearly badly, or that were likely acceptable for most purposes (Fig. 4). The other methods’ boundaries were not required to be perfect; we scored their output rather leniently. Our detailed scoring is presented in Supplementary Figs. 1–7.

The scene with widely varying cell sizes caused both YeastSpotter and Wood et al.’s method to make many mistakes (Fig. 4 top row). Generally, Wood et al.’s method tended to oversegment, i.e., subdivide cells erroneously. YeastSpotter tended to miss cells.

To find out how low the error rate of the YeaZ CNN may be, we analyzed the entire data sets 9 and 10 from ref. ^16^. The resulting segmentations were flawless for data set 9 for all 1596 cells except for four buds; tiny buds of a few pixels were detected early except for four (Supplementary Fig. 8), which were detected at the next time point when they were slightly bigger. The error rate is thus 0.25%. For data set 10, all 484 cells were segmented accurately (error rate: 0%); however, we remark that the images in data set 10 are very similar to each other.

Thus, on images from us and others that are challenging for other methods, YeaZ produced ground-truth level segmentations.

**Fig. 5:**
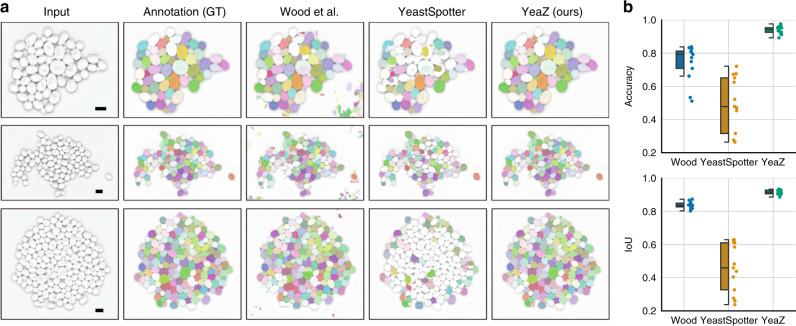
Detailed computational comparison of all methods. The evaluations were carried out on 17 test images of 1894 cycling wild-type cells not included in the YeaZ training set. **a** Each row shows an example test image, its ground-truth annotation (GT), and the result of Wood et al.^9^, YeastSpotter^14^, and YeaZ, respectively. **b** Quantification of segmentation performance of all methods. As is common in the computer vision literature, we call a predicted cell a true positive (TP), if its intersection over union (IoU) with the corresponding ground-truth (GT) cell is larger than or equal to 50%. Similarly, false positives (FPs) and false negatives (FNs) are defined as predictions that have no GT match and vice versa. As segmentation metric, we show the average accuracy ($\frac{\mathrm{TP}\,}{\mathrm{TP}\,+\mathrm{FP}\,+\mathrm{FN}\,}$) and average intersection-over-union of true positives (*IoU*). Boxes show interquartile ranges (IQR), lines signify medians, and whiskers extend to 1.5 IQR. Scale bar: 5 *μ*m.

To complement this analysis with a mathematical comparison, we also scored all three methods, YeaZ, YeastSpotter, and Wood et al., computationally. We took 17 semi-manually segmented phase contrast images containing 1894 wild-type cycling cells, which were not included in the training set for the YeaZ CNN, and computed standard segmentation metrics such as accuracy and mean intersection-over-union (IoU)^23^ (Fig. 5). The YeaZ CNN performed very well (mean accuracy: 94%) with most of the missed cells being small buds that the CNN delimited differently than the human annotators. Given that many of these buds spanned only a few pixels (see Supplementary Fig. 8 for examples of small buds), it was easy for two slightly different segmentations to differ by the 50% threshold for the accuracy metric—without the bud actually having been missed or clearly incorrectly segmented. By both metrics, YeastSpotter showed a substantially higher error rate than the other methods. Wood et al.’s method performed better than YeastSpotter on this set of images (mean accuracy: 79%). (Similarly, among the three test images in Fig. 4, Wood et al. had performed reasonably well for wild-type cycling cells (middle row).)

### Expanding the capabilities of the CNN

**Fig. 6:**
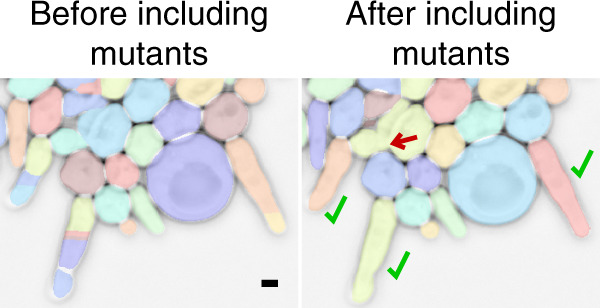
Adaptability of the CNN. clb1-6Δ mutants were either excluded (left) or included (right) in the training set for the CNN, which was then tested on an image of clb1-6Δ cells from a later time point with even longer filaments, shown here. Note that the color of each cell is dependent on the internal numbering and therefore arbitrary. However, there are no fragmented filamentous cells on the right (green check marks) although there are segmentation errors when the strangely shaped cells are crowded (red arrow). Phase contrast image inverted for better visualization. Scale bar: 2 *μ*m.

In order to gauge the adaptability of the CNN to new cell shapes, we trained it with and without approximately 50 filamentous clb1-6Δ cells growing in different colonies. We then tested the CNN on another image from a later time point of one of the scenes, when the filamentous cells had grown substantially longer (Fig. 6). Importantly, these longer cells were not broken up by the CNN trained on the expanded data set. Note that these colonies can be very difficult to segment by eye in the places where cells are crowded; thus, the mistakes that are made when strangely shaped mutant cells surround and partially overlap each other as in the bulk in Fig. 6 may be expected, given the number of clb1-6Δ mutants in the training set.

One solution to minimize the manual labor required to expand the training set is to proceed iteratively: segment a few images under a new condition, retrain the neural network with these images, and repeat with an improved neural network until the performance is acceptable.

### Graphical user interface (GUI)

**Fig. 7:**
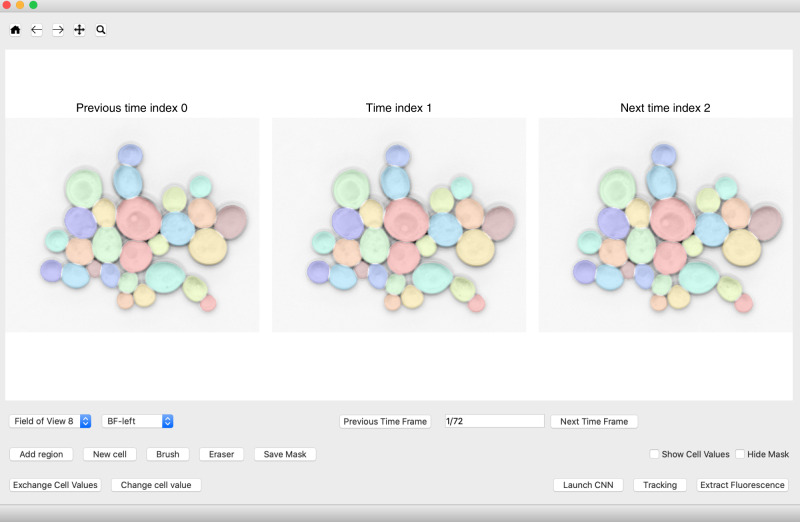
GUI for applying the YeaZ CNN, tracking cells, and correcting segmentations.

To apply the CNN and the tracking algorithm and correct their mistakes, we designed a Python-based GUI (Fig. 7). New cells can be drawn, modified after segmenting with the CNN, and cells can be relabeled. We were inspired by Microsoft Paint to include image manipulation tools such as brushes and erasers to manipulate the segmentation masks. The user can leaf through timelapse images with the current, the previous, and the next time point shown simultaneously, which can be helpful for verifying small buds. Fields of view and imaging channels can be changed. The GUI can read in multi-layer image files, folders of multiple image files, and Nikon ND2 files. We are continuously improving the capabilities of the GUI since we are using it ourselves. The latest version can be found through our website http://www.quantsysbio.com/data-and-software.

### Cell shapes reveal the timing and strength of morphogenesis control

**Fig. 8:**
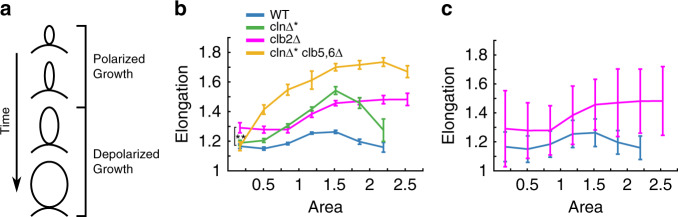
Clb2 promotes a more circular cell shape beginning early in the cell cycle. **a** Schematic of cell shape during growth. **b** Mean and SEM of elongation versus area for different cyclin mutants. **: *p* = 0.004, single-tail t-test. **c** Mean and STD for two of the populations from panel **b** illustrating the variability in the data. **b**, **c** Abscissa scaled so that 1 is the mean area of wild-type cells, corresponding to 16.3 *μ*m^2^. *n*=525 (WT), 500 (*cln*Δ^*^), 597 (*clb2*Δ), 543 (*cln*Δ^*^clb5,6Δ). The largest 2.5% of the overall population was discarded. The remaining range was binned into 8 equal intervals. There were no WT or *cln*Δ^*^ cells in the largest bin, therefore, no values are shown.

New yeast daughter cells grow as buds from the tip until mitotic cyclins, mainly Clb2, change the direction of growth from apical to isotropic (Fig. 8a); overexpressing the cell cycle Start initiator *CLN2* or deleting *CLB2* leads to more elongated cells^26,27^. Since Clb2 turns on as part of a positive feedback loop some time after cell cycle Start^28^, one may expect growth depolarization to occur suddenly at a specific time after budding. To investigate when this switch occurs, we analyzed the geometries of hundreds of wild-type and mutant cells using YeaZ (Fig. 8b, c). We quantified each cell’s elongation (=major axis/minor axis) by equating its second moments of the area with those of an ellipse. Based on images taken at single time points only, we used the cells’ areas as stand-ins for the time after budding because cells grow in size continuously.

Wild-type cells (blue) initially became more elongated with size, and depolarization kicked in when cells reached around 1–1.5 of the mean wild-type area, making cells more circular again (Fig. 8b).

*cln1-3*Δ*MET3pr-CLN2* (abbreviated: *cln*Δ^*^) cells (green) which expressed the Start cyclin *CLN2* continuously in medium lacking methionine started as buds that were similarly shaped as wild type but became substantially more elongated (Fig. 8b). Subsequently, however, they depolarized and grew sufficiently to end up about as circular as wild-type cells when they were large.

Interestingly, *clb2*Δ cells (magenta), were already substantially more elongated when they were very small (first bin: 0-33% of mean wild-type area), even though Clb2 seemed to depolarize wild-type cells much later at around 1–1.5 mean wild-type area (compare wild-type and *clb2*Δ in Fig. 8b). Thus, Clb2 influenced cell morphology already very early in the cell cycle, potentially because of i) early, weak activation of *CLB2*, ii) basal expression of *CLB2*, iii) left-over Clb2 from the previous cycle, or iv) a *CLB2*-dependent remnant from the previous cell cycle. Furthermore, *clb2*Δ cells could not be detected to depolarize at all, and became even more rod-like with size.

To test whether Clb2 is initiated at low levels earlier than previously thought, we analyzed *cln*Δ^*^clb5,6Δ (yellow) cells (Fig. 8b). *CLB5* and *CLB6* are key activators of *CLB2*^28^. We combined their deletions with the *cln*Δ^*^ mutations and constructs, which produce Cln2 continuously in minus methionine medium. This was done to maximize polarized growth, which Cln2 promotes, and compensate for the loss of Clb5, which might also promote polarized growth as it can substitute for cell cycle Start initiators. Nevertheless, cells started similarly shaped as wild-type or *cln*Δ^*^ cells, inconsistent with explanation i). Explanations ii) and iii) would be surprising because Clb2 is known to interfere with origin of replication licensing in G1 phase^29^, before cell cycle Start; however, ii) or iii) would be consistent with the requirements for origin of replication licensing if licensing is less sensitive to Clb2 than morphogenesis control.

In summary, our data refine our understanding of the timing and strength of morphogenesis control, to be earlier than commonly thought and to be gradually strengthening with time, not simply switching on-off. Both results are surprising considering the known timing and manner of activation of Clb2. Since we focused primarily on the geometry of the smallest cells, any potential minor differences in growth rates between the mutants should not affect our conclusions.

This application exemplifies why an efficient segmentation method is needed and how it can provide new insights. Because variability is high (see standard deviation in Fig. 8c), large numbers of cells are needed for statistically significant results (see standard error of the mean in Fig. 8c).

## Discussion

We present a freely available, large, diverse, and high-quality set of segmented yeast images as well as a CNN trained on this data set. The CNN segments new images recorded by us and others very accurately. We introduced a simple cell-cell boundary test to alleviate the oversegmentation problem that arises in the absence of an established unique interior point, which a fluorescent nuclear marker provides in other methods^17^. Our approach does not require extra fluorescent markers.

There is a body of work on correcting oversegmentation^18–21^. Interestingly, an idea similar to ours, namely, that a boundary is artefactual if the average of the boundary pixels’ CNN scores is close 1, which means that those boundary pixels are actually cell-like, was considered but not pursued further^18^. The reason was that erroneous boundaries may include real boundary pixels (with CNN scores ≈ 0) at their two ends which make the averaged CNN score ambiguous. We circumvent this problem by simply ignoring the bottom 1/4 of lowest-scoring pixels and averaging over the top 3/4, thereby, ignoring the two ends of any artefactual borders. This straightforward fix may work well for microscopy images of many microbes because the image resolution is generally sufficiently high compared to the geometric features in the interiors of cells; enough evidence can be gathered about whether a border is fake or real since artefactual borders will be made up of many pixels. For small cells, where this would not be the case, we suppress oversegmentation by forbidding too many close-by seeds. Thus, images of many microbes may allow simpler approaches than macroscopic objects^18^.

Our CNN-based analysis suggests that basal *CLB2* expression, left-over Clb2, or Clb2-dependent signals from the previous cell cycle influence cell shape early in a new cycle, not just when cells depolarize markedly. The influence of Clb2 early in the cell cycle is surprising and, to our knowledge, has not been observed previously.

While we designed our data set to be sufficiently diverse for most applications, there may arise conditions under which it is not. Should the CNN perform poorly for certain new cell shapes or conditions, in principle, adding challenging semi-automatically segmented training examples to the current set ought to improve the performance, as we demonstrated for clb1-6Δ cells, or perfect it. Repeated cycles of segmenting with an incrementally improving CNN, correcting mistakes, and retraining may be a particularly labor-efficient way to expand the capabilities of the CNN.

As a proof of principle for how the existing CNN can be leveraged to improve it further, we applied a simple trick to expand the training set beyond phase contrast images: We recorded the same scene with both phase contrast and bright-field microscopy and used the CNN to segment the phase contrast images. This gave us a training set for bright-field images with little effort. We make the bright-field segmentations available although we did not train the CNN with it.

## Methods

### Images and imaging conditions

Recordings were made with a 60x objective and a Hamamatsu Orca-Flash4.0 camera. Cells were grown in CellASIC microfluidic chips in standard synthetic complete (SC) medium supplemented with different sugars, glucose, galactose, or raffinose, depending on the experiment. Images have 16 bit depth. The diascopic light was generated by Nikon Ti2-E LEDs. Exposure times were 100 ms. We varied light intensities such that in the training set, median pixel intensities ranged from 169 to 1329 (bottom to top 2% of images) and the contrast in each image (bottom to top 2% of pixels divided by median) ranged from 1.4 to 3.7 (bottom to top 2% of images).

### Pre-processing

The training set consists of (i) microscopy images and (ii) mask images from the semi-manual annotation (see ‘Data set’) which are of the same size as the microscopy images and whose pixels denote the ID numbers of the cells in the corresponding microscopy images. Background pixels correspond to 0 in the masks. Before setting all cell numbers to 1 for training the neural network, we found the borders between different cells by dilating each cell and identifying intersecting pixels. These border pixels were then also set to 0 in the mask images. The training set was cut into 256 x 256 images, which overlapped by at least half in width or height, for the training. (The GUI applies the CNN to whole images without cropping.)

### Training

We downloaded the U-net implementation from https://github.com/zhixuhao/unet and adapted it. Batch sizes were set to 25 and training was carried out for 100 epochs. Augmentation was performed with rotation range 90°, shear range 45°, zoom range 0.5–2, horizontal and vertical flipping, and brightness range 0.5–1.5.

### Evaluation of tracking

We corrected cell ID numbers manually for one of the timelapse recordings (a_reexport1_crop_1) and used it to evaluate the tracking method.

### Data analysis

Data analysis was performed in Matlab R2018b.

### Strains

All strains were W303 based. All except AS18 have been characterized previously^30,31^. See strain list in Supplementary Table 3.

### Reporting summary

Further information on research design is available in the Nature Research Reporting Summary linked to this article.

## Supplementary information

Supplementary Information

Reporting Summary

## Data Availability

All segmentation data sets can be downloaded through our website: http://www.quantsysbio.com/data-and-software.

## Code availability

The latest version of the YeaZ GUI can be downloaded through our website, where the CNN can also be tested online: http://www.quantsysbio.com/data-and-software. The YeaZ GUI is directly available at: github.com/lpbsscientist/YeaZ-GUI.
