# Supplementary material for: A convolutional neural network segments yeast microscopy images with high accuracy
Source: Nat Commun. 2020 Nov 12;11:5723. doi: 10.1038/s41467-020-19557-4 (PMC7665014; doi:10.1038/s41467-020-19557-4)
Supplement: Supplementary file 1 — Supplementary Information [file 41467_2020_19557_MOESM1_ESM.pdf]

Supplementary Material

A convolutional neural network segments yeast microscopy images with high accuracy

Dietler et al.

Supplementary Figures

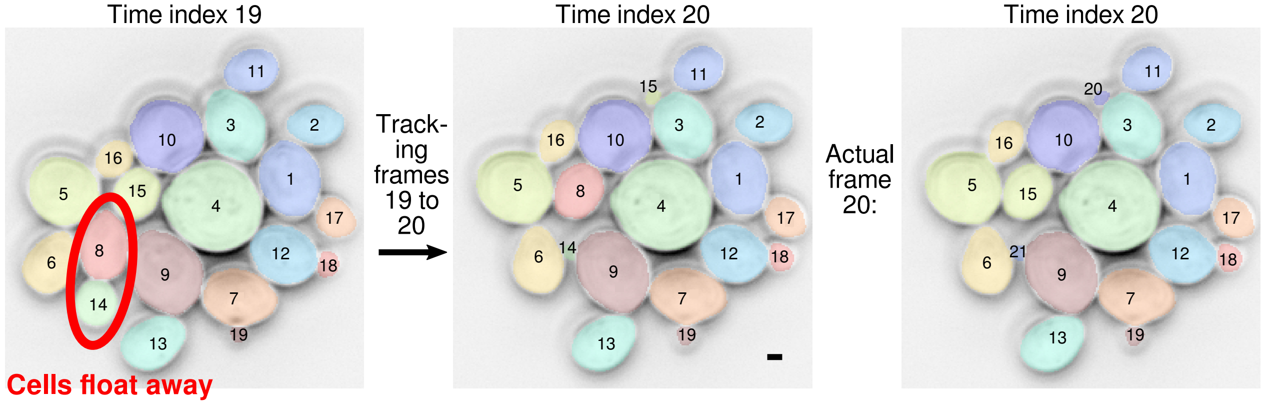

Supplementary Figure 1: Tracking errors caused by two cells floating out of the field of view. Scale bar: 1 μm.

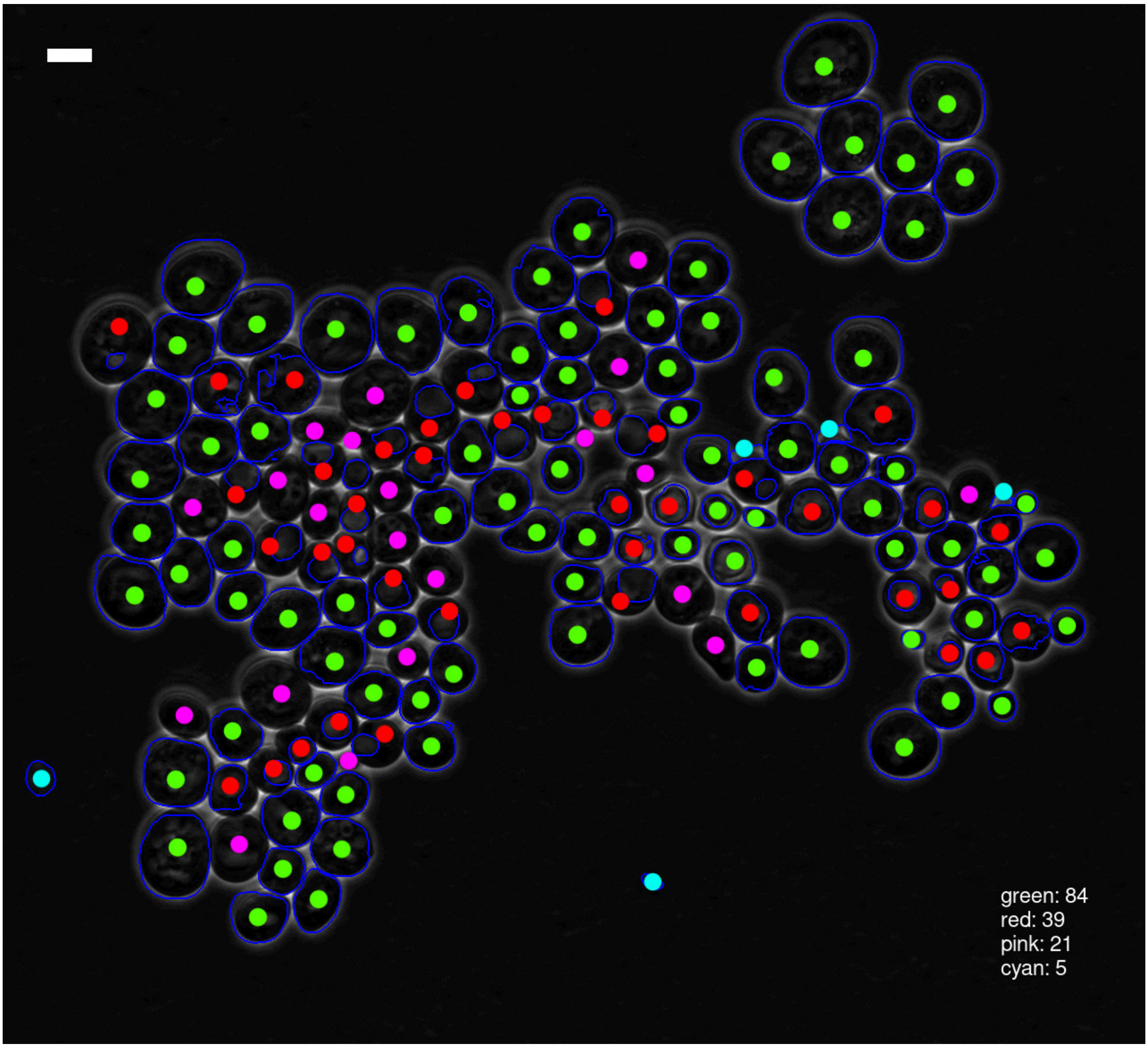

Supplementary Figure 2: Scoring of YeastSpotter results. Green dots: acceptable segmentation, red: bad segmentation, pink: missed cell, cyan: spurious cell. Scale bar: 5 μm.

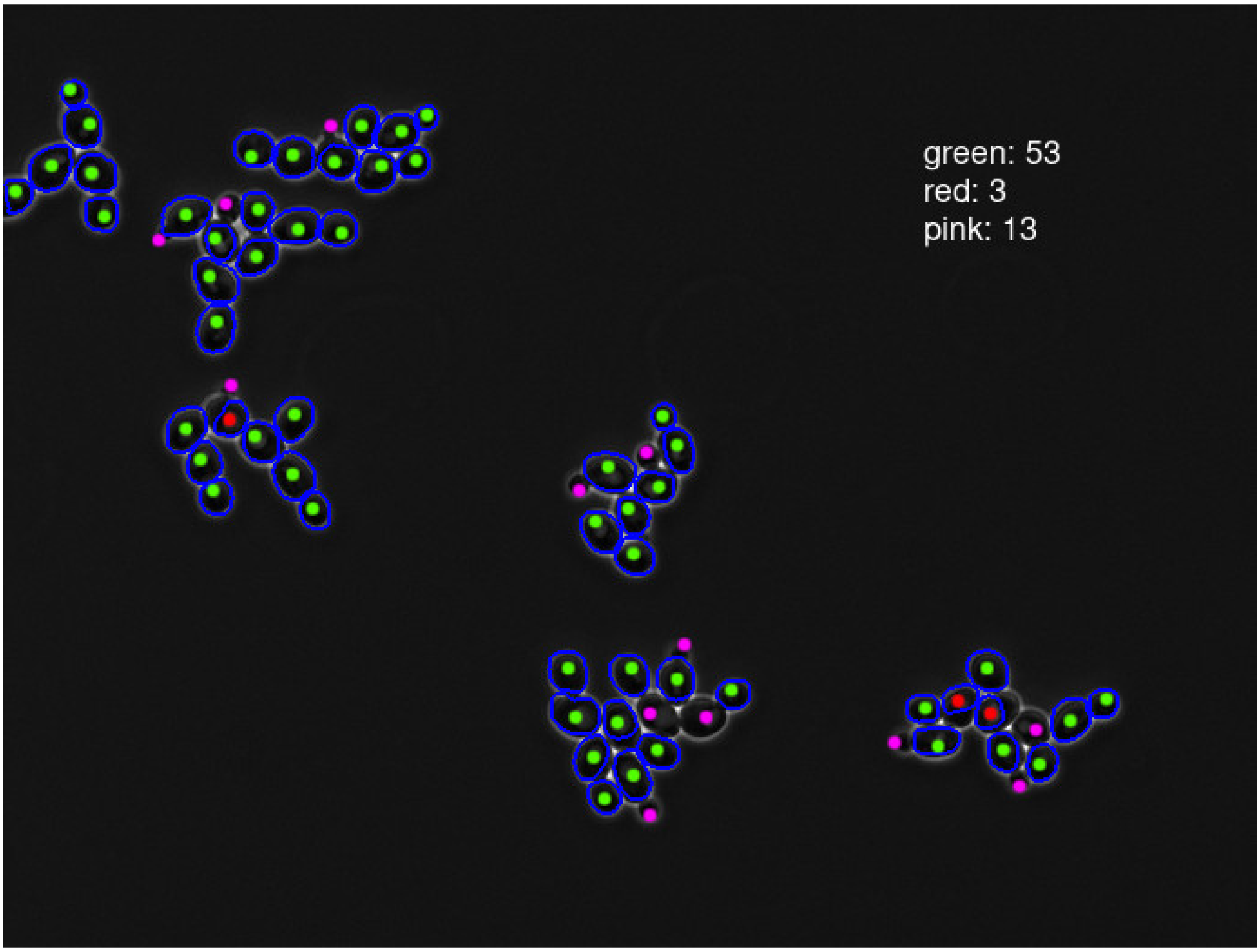

Supplementary Figure 3: Scoring of YeastSpotter results. Green dots: acceptable segmentation, red: bad segmentation, pink: missed cell, cyan: spurious cell. Image from ref. (1), scale bar unknown.

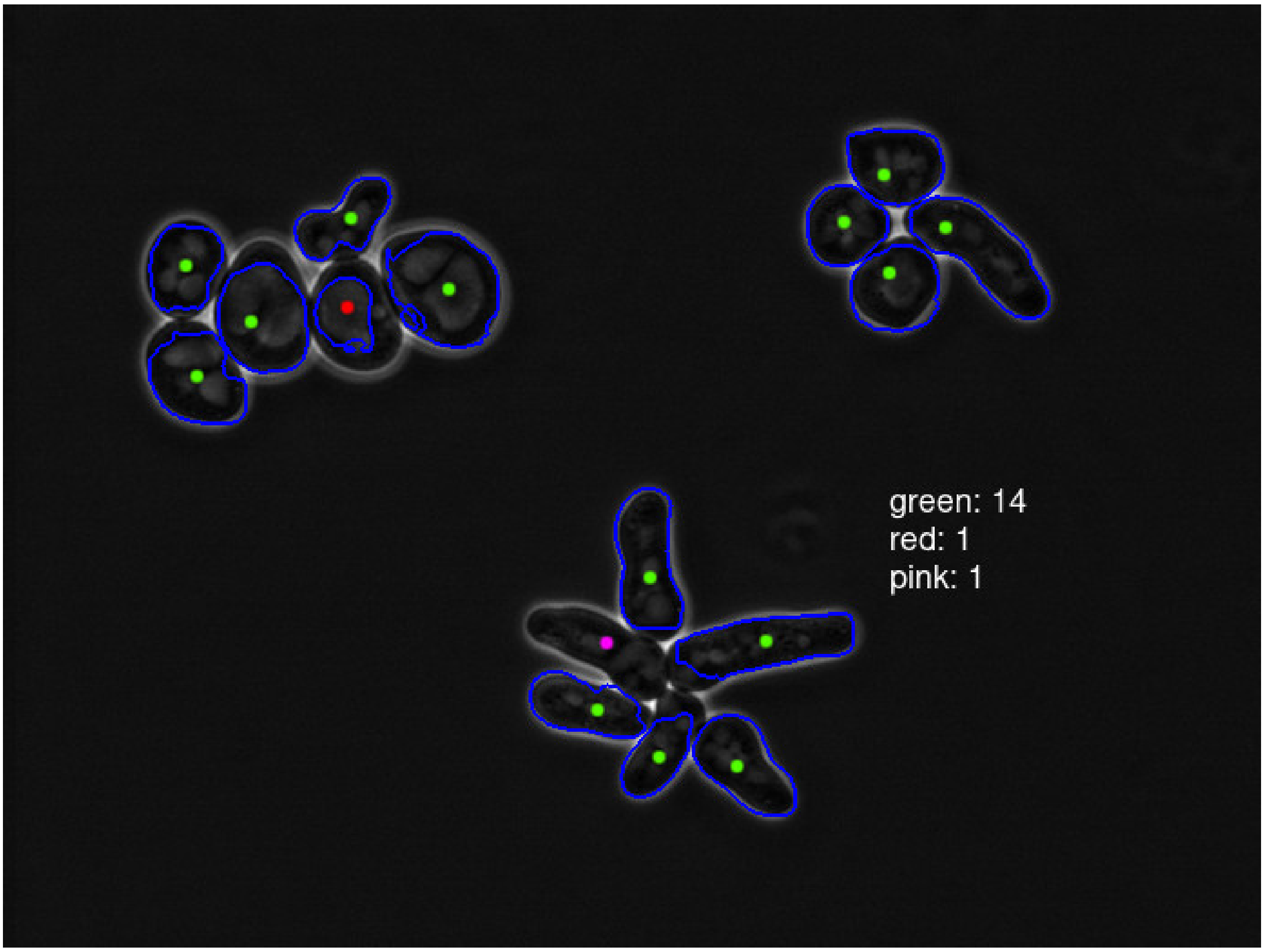

Supplementary Figure 4: Scoring of YeastSpotter results. Green dots: acceptable segmentation, red: bad segmentation, pink: missed cell, cyan: spurious cell. Image from ref. (1), scale bar unknown.

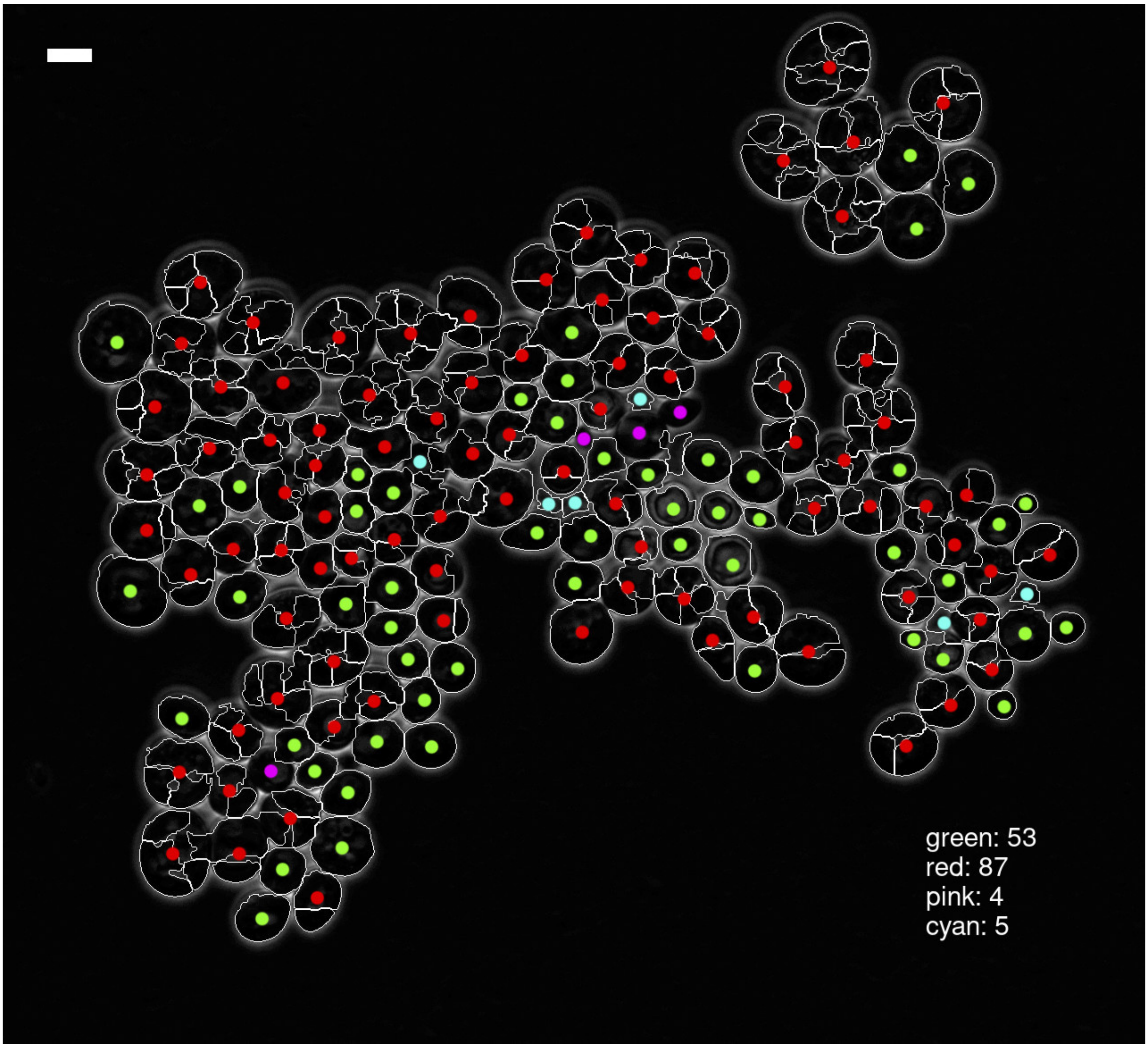

Supplementary Figure 5: Scoring of the results of the method by Wood et al. (2). Green dots: acceptable segmentation, red: bad segmentation, pink: missed cell, cyan: spurious cell. Scale bar: 5 μm.

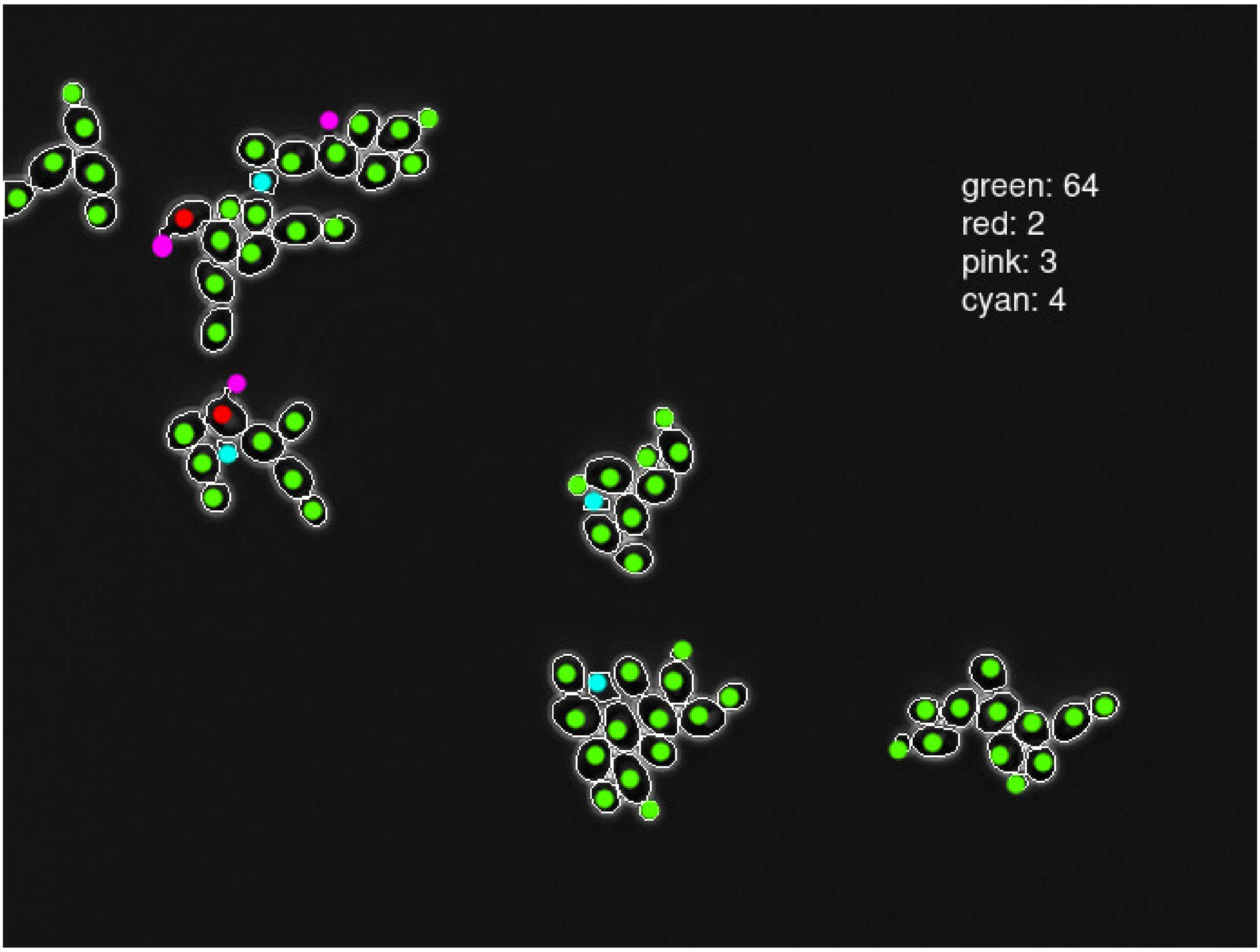

Supplementary Figure 6: Scoring of the results of the method by Wood et al. (2). Green dots: acceptable segmentation, red: bad segmentation, pink: missed cell, cyan: spurious cell. Image from ref. (1), scale bar unknown.

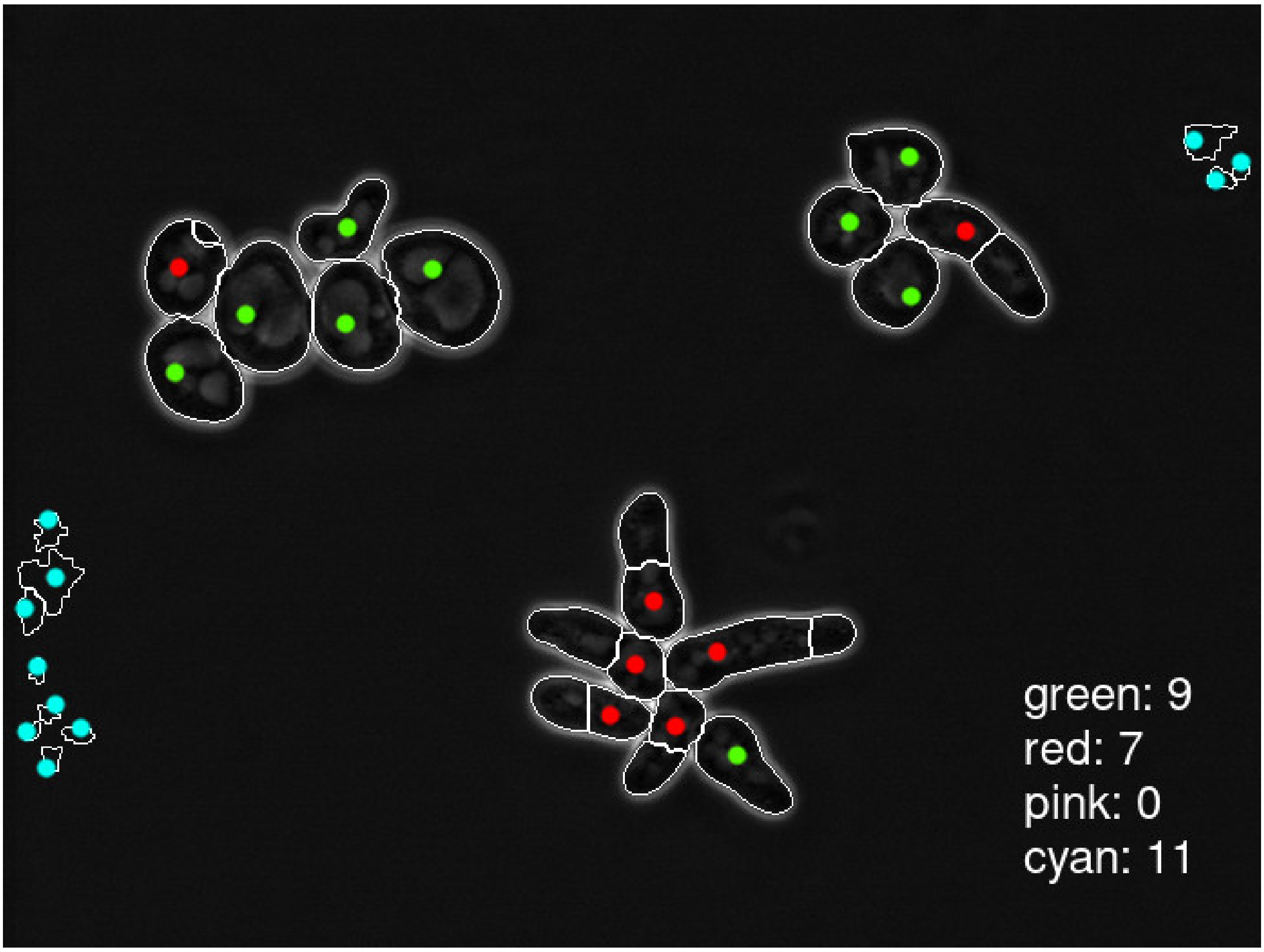

Supplementary Figure 7: Scoring of the results of the method by Wood et al. (2). Green dots: acceptable segmentation, red: bad segmentation, pink: missed cell, cyan: spurious cell. Image from ref. (1), scale bar unknown.

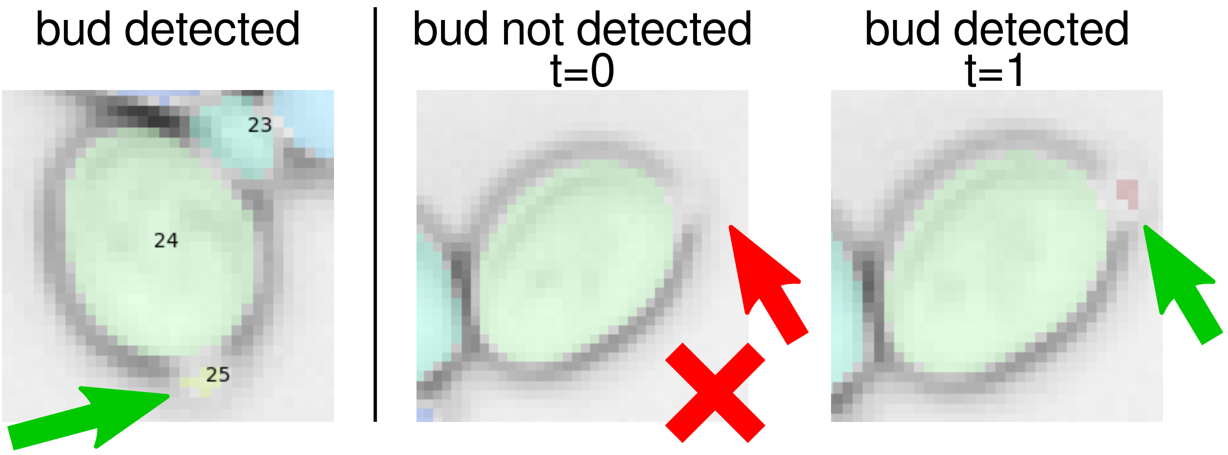

Supplementary Figure 8: Examples of tiny buds in data set 9 from ref. (1), some only a few pixels in size, that were detected by the YeaZ CNN (left and right) except for four such tiny buds (middle example). The missed bud (middle) was, however, detected at the next time point in the timelapse series (right) when it was slightly bigger. Image from ref. (1), scale bar unknown.

Supplementary Tables

Supplementary Table 1: Training set images

File name	Number of image layers	Number of cells
a_reexport1_crop_1	75	1914
a_reexport2_crop_1	42	703
a_reexport3_crop_1	41	132
a_reexport3_crop_2	2	0
d_reexport1_crop_1	30	110
d_reexport1_crop_2	30	764
d_reexport1_crop_3	30	412
d_reexport1_crop_4	1	30
d_reexport2_crop_1	1	20
d_reexport2_crop_2	30	345
d_reexport2_crop_3	30	611
m_reexport1_crop_1	18	1637
v_cdc20null_crop_1	1	171
v_clnnull_crop_1	1	107
Ahmad_frame_16_crop_1	5	251
Ahmad_frame_16_crop_2	5	18
Ahmad_frame_16_crop_3	5	10
Ahmad_frame_16_crop_4	5	247
Ahmad_frame_19_crop_1	5	208
Ahmad_frame_19_crop_2	5	158
Ahmad_frame_19_crop_3	5	135
V11032020_p1_crop_1	1	27
V11032020_p2_crop_1	4	200
V11032020_p3_crop_1	2	105
2020_3_24_AS20_1_AS18_Pos11_crop_1	5	137
2020_3_24_AS20_1_AS18_Pos11_crop_2	5	76
Total	384	8528

Supplementary Table 2: Bright-field image segmentations

File name	Number of image layers	Number of cells
FOV2_1.5p_PhC_absent_crop_1	1	97
FOV3_1.5p_PhC_absent_crop_1	1	83
FOV4_1.5p_PhC_absent_crop_1	1	83
FOV5_1.5p_PhC_absent_crop_1	1	47
FOV6_1.5p_PhC_absent_crop_1	1	107
FOV7_1.5p_PhC_absent_crop_1	1	100
FOV8_1.5p_PhC_absent_crop_1	1	129
FOV9_1.5p_PhC_absent_crop_1	1	58
FOV9_1.5p_PhC_absent_crop_2	1	206
FOV10_1.5p_PhC_absent_crop_1	1	274
FOV10_1.5p_PhC_absent_crop_2	1	62
FOV11_1.5p_PhC_absent_crop_1	1	62
FOV12_1.5p_PhC_absent_crop_1	1	181
FOV13_1.5p_PhC_absent_crop_1	1	74
FOV14_1.5p_PhC_absent_crop_1	1	105
FOV15_1.5p_PhC_absent_crop_1	1	62
Total	16	1730

Supplementary Table 3: Strains used.

Name	Mating type locus	Genotype
AX42-25A	MATa	wild type W303
JB204-6B	MATa	ADE2 HTB2-mCherry::HIS5
SJR112a12d	MATα	cln1Δ0 cln2Δ0::MET3pr-CLN2 cln3Δ0 HTB2-mCherry::HIS5 ura3Δ0
SJR33h6d		clb2Δ::LEU2 trp1Δ::TRP1::GAL-SIC1(1X) HTB2-mCherry:HIS5 MYO1-GFP::KanMX
SJR36a11b	MATa	cln1Δ cln2Δ:CLN2pr-Venus:TRP1 cln3Δ:LEU2 trp1Δ:TRP1:MET3pr-CLN2 clb5Δ:HIS3 clb6Δ:KanMX HTB2-mCherry:HIS5
CL86-1-11D	MATa	clb1Δ clb2Δ::GALL-CLB2::URA3 clb3Δ::TRP1 clb4Δ::his3Δ::kanMX clb5Δ::kanMX clb6Δ::kanMX swe1Δ::TRP1 ADE2
2147-1C	MATα	cdc20Δ::LEU2 ADE2::GALL-CDC20
AS18	MATαsyn	cln1Δ0 cln2Δ0::MET3pr-CLN2 cln3Δ0::GAL1pr-HO HTB2-mCherry::HIS5 ADH1pr-HOcs-YFP

References

(1) Mróz, F., Kaczmarek, A. & Stoma, S. YIT - Yeast Image Toolkit. *http://yeast-image-toolkit.biosim.eu/pmwiki.php* (accessed: 15.02.2020).

(2) Wood, N. E. & Doncic, A. A fully-automated, robust, and versatile algorithm for long-term budding yeast segmentation and tracking. *PLoS One* **14**, 1–28 (2019).
