# Supplementary material for: A convolutional neural network segments yeast microscopy images with high accuracy
Source: Nat Commun. 2020 Nov 12;11:5723. doi: 10.1038/s41467-020-19557-4 (PMC7665014; doi:10.1038/s41467-020-19557-4)
Supplement: Supplementary file 2 — Reporting Summary [file 41467_2020_19557_MOESM2_ESM.pdf]

nature research

Corresponding author(s): Rahi, Sahand Jamal

Last updated by author(s): Oct 14, 2020

Reporting Summary

Nature Research wishes to improve the reproducibility of the work that we publish. This form provides structure for consistency and transparency in reporting. For further information on Nature Research policies, see our Editorial Policies and the Editorial Policy Checklist.

Statistics

For all statistical analyses, confirm that the following items are present in the figure legend, table legend, main text, or Methods section.

n/a	Confirmed	
☐	☒	The exact sample size (*n*) for each experimental group/condition, given as a discrete number and unit of measurement
☐	☒	A statement on whether measurements were taken from distinct samples or whether the same sample was measured repeatedly
☐	☒	The statistical test(s) used AND whether they are one- or two-sided<br>*Only common tests should be described solely by name; describe more complex techniques in the Methods section.*
☒	☐	A description of all covariates tested
☒	☐	A description of any assumptions or corrections, such as tests of normality and adjustment for multiple comparisons
☐	☒	A full description of the statistical parameters including central tendency (e.g. means) or other basic estimates (e.g. regression coefficient) AND variation (e.g. standard deviation) or associated estimates of uncertainty (e.g. confidence intervals)
☐	☒	For null hypothesis testing, the test statistic (e.g. *F*, *t*, *r*) with confidence intervals, effect sizes, degrees of freedom and *P* value noted<br>*Give P values as exact values whenever suitable.*
☒	☐	For Bayesian analysis, information on the choice of priors and Markov chain Monte Carlo settings
☒	☐	For hierarchical and complex designs, identification of the appropriate level for tests and full reporting of outcomes
☒	☐	Estimates of effect sizes (e.g. Cohen's *d*, Pearson's *r*), indicating how they were calculated

Our web collection on statistics for biologists contains articles on many of the points above.

Software and code

Policy information about availability of computer code

Data collection	Custom software was used to segment images for the training set. The resulting segmentations are part of this publication and can be inspected by the reader.
Data analysis	Data analysis was performed in Matlab R2018b using standard Matlab functions.

For manuscripts utilizing custom algorithms or software that are central to the research but not yet described in published literature, software must be made available to editors and reviewers. We strongly encourage code deposition in a community repository (e.g. GitHub). See the Nature Research guidelines for submitting code & software for further information.

Data

Policy information about availability of data

All manuscripts must include a data availability statement. This statement should provide the following information, where applicable:

- Accession codes, unique identifiers, or web links for publicly available datasets
- A list of figures that have associated raw data
- A description of any restrictions on data availability

Data is freely available through our website http://www.quantsysbio.com/data-and-software .

Field-specific reporting

Life sciences study design

All studies must disclose on these points even when the disclosure is negative.

Sample size	Given the efficiency of the YeaZ segmentation system, we segmented a large number of cells at once. Because the results were statistically significant, we stopped. The sample size was not predetermined. We tried to segment as diverse and as many yeast images as reasonably possible.
Data exclusions	As noted in the caption of Fig. 8, we discarded the largest 2.5% of cells because they interfered with reasonable binning. Since this was done for the whole data set, i.e., across all genotypes, this measure implied that our results apply to 97.5% of cells generally. If we tried to include all cells in our bins, we would have had to extend bins for very few additional points or add bins with very few cells.
Replication	We performed the measurements for Fig. 8 on hundreds of cells recorded at the same time. Fig. 8 was only performed once.
Randomization	Since the images were analyzed fully automatically by the YeaZ system we present here, randomization was not necessary.
Blinding	Segmentation was performed automatically by our YeaZ system

Reporting for specific materials, systems and methods

We require information from authors about some types of materials, experimental systems and methods used in many studies. Here, indicate whether each material, system or method listed is relevant to your study. If you are not sure if a list item applies to your research, read the appropriate section before selecting a response.

Materials & experimental systems

n/a	Involved in the study	
☒	☐	Antibodies
☒	☐	Eukaryotic cell lines
☒	☐	Palaeontology and archaeology
☒	☐	Animals and other organisms
☒	☐	Human research participants
☒	☐	Clinical data
☒	☐	Dual use research of concern

Methods

n/a	Involved in the study	
☒	☐	ChIP-seq
☒	☐	Flow cytometry
☒	☐	MRI-based neuroimaging
